# Improving cataract services in the Indian context

**Published:** 2014

**Authors:** Gudlavalleti VS Murthy, BK Jain, BR Shamanna, D Subramanyam

**Affiliations:** Director: Indian Institute of Public Health (Public Health Foundation of India), Hyderabad, India. murthy.gvs@iiphh.org; Trustee and Director: Shri Sadguru Seva Sangh Trust & Sadguru Netra Chikatsalaya, Chitrakoot, India.; Associate Professor: School of Medical Sciences, University of Hyderabad, Hyderabad, India.; Head: Community Ophthalmology Programmes, Sadguru Netra Chikatsalaya, Chitrakoot, India.

**Figure:**
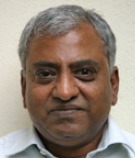
Gudlavalleti VS Murthy

**Figure:**
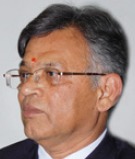
BK Jain

**Figure:**
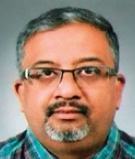
BR Shamanna

**Figure:**
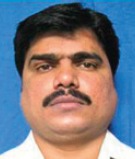
D Subramanyam

In many countries, the number of cataract operations performed is inadequate to deal even with the people who have newly become blind from cataract, let alone those who are already blind or visually impaired. There is, therefore, a backlog of cases needing surgery. This could be due to low surgical capacity (people are on a waiting list) or to a lack of demand for cataract surgery (people haven't come forward for the services they need and there is therefore no waiting list).

India has been very successful in raising its cataract surgical rate (the number of operations per million people, per year), from just over 700 in 1981, to 6,000 in 2012.^1^, ^2^ This is much closer to the estimated cataract surgical rate of 8,000–8,700 needed to eliminate blindness due to cataract in India.^3^ Much of this is the result of increased efficiency, with surgeons being able to perform twenty operations per day thanks to innovations in surgical technique, good team work with appropriate staffing levels, use of day case surgery, and improvements in operating theatre design.^4^

In order that people can come for surgery in large numbers, however, demand for cataract surgery must be created in the community. This means that:

1. People must be **aware.** They must know that the condition they have is cataract, that surgery gives good results, and where to go for surgery.
2. People must have **access.** There must be services available within reach, family members must be willing to support or allow the person to undergo cataract surgery, and any other barriers to attending for surgery must be successfully addressed (e.g. for people with disabilities or women).
3. People must be able to **afford** cataract surgery, including any associated cost (e.g. for transport).

**Figure:**
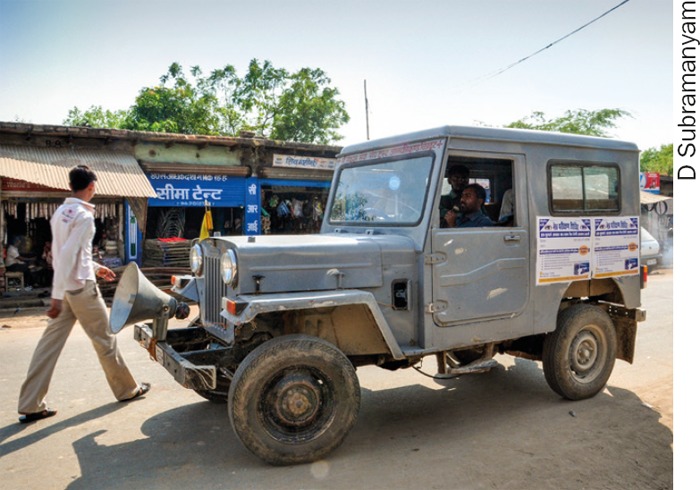
This vehicle helps to deliver educational materials during outreach camps

Finally, patients and their family must be confident in the quality and safety of surgery.

In India, social marketing approaches were used to create awareness of cataract. This included addressing beliefs about causation (e.g. that it is an ‘act of god’) and improving people's understanding that cataract is a common occurrence in older people and that it can be treated. Better quality services also helped to enhance the credibility of cataract surgery and to improve people's confidence in the services being offered.

By identifying barriers such as cost, distance, or lack of an accompanying person, and by providing appropriate solutions (such as subsidies, transportation, and practical support for accompanying persons), service providers in India were able to prioritis hard-to-reach populations including women, tribal populations and the poor. A ‘menu’ of service options was provided and individuals were allowed to choose services based on their ability to pay.

The non-governmental organisation (NGO) sector and Indian ophthalmologists have continued to experiment with innovative approaches to increasing demand for cataract services, many of which have been adopted by countries in the rest of the world. Different approaches include outreach camps to detect those needing surgery, use of counsellors to explain the procedure and what to expect, tiered pricing mechanisms, and local production of surgical consumables and equipment.

Sadguru Netra Chikatsalaya (SNC), Sri Sadguru Seva Sangh Trust, Chitrakoot, is located in a remote and economically deprived region in Central India. It is one example of a hospital which has been able successfully to create demand for cataract surgery even amongst the hard-to-reach, and has also made surgery affordable. The feudal nature and socio-economic deprivation of this region are well known.

This article aims to show how the hospital has:

- created a demand in the community for cataract services, even in the summer
- reached the hard-to-reach
- approached the cost and affordability of surgery.

## What has changed?

SNC has evolved from a makeshift surgical camp, only operational during the winter months (a time when communities thought surgery to be safer), to a permanent, high-volume, high-quality and affordable facility. A 350-bed eye hospital was built in 2004, following an innovative planning exercise undertaken in 2002.

Although the initial plan was to provide services at no cost to patients, during the planning exercise it became clear that SNC would have to generate income from patients in order to ensure long term sustainability. Table 1 shows how things have changed.

Outreach screening camps now take place in ten districts in Uttar Pradesh and six districts in Madhya Pradesh and follow a very regular schedule, so communities know when the camps will take place at any given location. The eye hospital serves a total population of nearly 50 million people. Primary eye care has been strengthened through the establishment of 26 vision centres. New construction is supported by NGOs while free surgery and related logistics are supported by the District Blindness Control Society. All other running costs are borne by the hospital itself. All operations are performed at the base hospital and transportation to the hospital and back is provided at no cost to the patients. Post-operative follow-up is done at the nearest vision centre and at the next outreach camp to take place in that area.

**Table 1:** Outcome of the planning exercise undertaken in 2002

Before the planning exercise	2013
Rapid turnover of skilled human resources – difficult to attract ophthalmologists	60 full time ophthalmologists and dedicated administrative support staff
23,525 cataract operations per year	117,543 cataract operations per year
45% intra-ocular lens (IOL) implants	99% IOL implants
12% cataract surgery during the summer months	35% of cataract surgery during the summer months
65% free surgery; 34% subsidised; 1% of patients paying the full cost	43% free surgery; 42% subsidised; 15% of patients paying the full cost
Predominantly dependent on non-governmental organisations and philanthropists for running costs. The District Blindness Control Society and philanthropists paid for free surgery.	All running costs covered by patient fees. Free surgery supported by grants from the District Blindness Control Society

**Figure:**
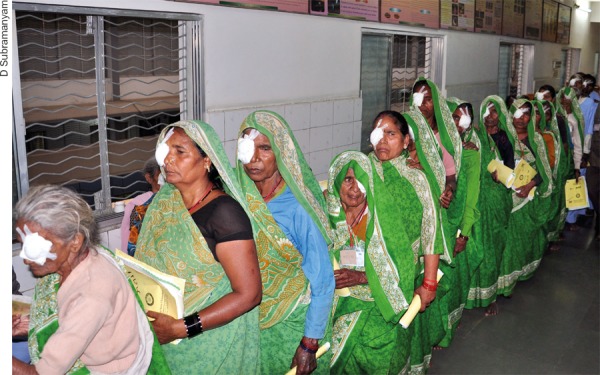
Operated patients leaving the operating theatre

The outcome of these initiatives is reflected in the increase in service delivery (Table 2). This illustrates how cataract services can be made accessible, acceptable and affordable even in a very difficult and hard-to-reach area where there is a lack of providers. This hospital is an example of how appropriate needs-based cataract services and a growth trajectory can be achieved and sustained with good governance and a responsive leadership team.

## How did these changes come about?

To overcome the barriers of seasonal imbalance, the hospital management initiated an outreach programme in the summer months. Free eye check-ups, free transportation and free diet and counselling services motivated people to accept the services and this led to a gradual increase in the volume of patients.

The outreach team struggled during the first three years, as the concept of surgery during the summer months was totally new in the region. To raise awareness of the services offered, SNC distributed educational materials, organised regular meetings with influential members of the community and engaged the local community in key events at the hospital.

Counselling of patients was focused on the improvements in cataract surgery techniques, i.e. IOL implantation.

Another challenge was to ensure that women, the poor and people with disabilities also came forward for surgery. A variety of strategies were used.

- Camps were organised in remote rural locations to bridge the gap between the hospital and community.
- Poor and needy people were provided with free transportation, medicines, examinations, dietary advice and surgery.
- Regular orientation and education sessions were organised for the heads of families to encourage them to increase the uptake of services among the women in their family.
- People accompanying those who were bilaterally blind, those with one eye, and those who were disabled were offered free transport, food and accommodation.
- One or two volunteers from each area visited during outreach were encouraged to accompany their community members to the hospital for surgery. This reduced indirect costs to the families. Accommodation and food was also provided to volunteers and escorts who returned the patients home after discharge.

**Table 2:** Outomes of the innovative approaches implemented at SNC Chitrakoot, India

Parameters	2002	2013	% change
Out patients registered	97,304	549,220	464%
Surgeries performed	29,315	117,543	301%
Number of outreach camps	42	547	1202%
Operational cost recovery	78%	100%	
IOL Surgery	55%	99%	80%

To reduce fear, which affects people's willingness to accept surgery, SNC adopted the following approaches:

- using a model (dummy) lens during counselling to explain to patients about IOL implants etc.
- using the local language or dialect to counsel the patients
- explaining the facilities available in the hospital
- allowing family members to travel with the patient
- sharing the stories of people from the same community who had been successfully operated on. (Hearing about someone's increased productivity following surgery can also encourage families to support a family member to accept surgery.)

Today, SNC is engaged in providing outreach services through ten outreach teams and 26 vision centres within a 250 km radius of the hospital The increase in outreach camps did not have a negative impact on the number of walk-in patients at SNC. There has been a parallel growth in the number of operations done as a result of outreach camps and those done on walk-in patients.

## What can we learn from SNC?

For this model to work elsewhere, there needs to be proper programme management, effective planning and utilisation of resources, and a periodic review of strategies. In the area served by SNC, the barriers related to awareness, access and affordability are very similar to those in other countries where access to services is poor. We have shown that they can be overcome through perseverance and by using a strategic approach.
